# IGDD: a database of intronless genes in dicots

**DOI:** 10.1186/s12859-016-1148-9

**Published:** 2016-07-27

**Authors:** Hanwei Yan, Xiaogang Dai, Kai Feng, Qiuyue Ma, Tongming Yin

**Affiliations:** 1 Key Laboratory of Forest Genetics & Biotechnology, Nanjing Forestry University, Nanjing, China; 2 Laboratory of Modern Biotechnology, Anhui Agricultural University, Hefei, China

**Keywords:** Intronless genes, Database, Dicots

## Abstract

**Background:**

Intronless genes are a significant characteristic of prokaryotes. Systematic identification and annotation are primary and crucial steps for determining the functions of intronless genes and understanding their occurrence in eukaryotes.

**Description:**

In this paper, we describe the construction of the Intronless Genes Database in Dicots (IGDD; available at http://bio.njfu.edu.cn/igdd/), which contains data for five well-annotated plants including *Arabidopsis thaliana*, *Carica papaya*, *Populus trichocarpa*, *Salix suchowensis* and *Vitis vinifera*. Using highly visual settings, IGDD displays the structural and functional annotations, the homolog groups, the syntenic relationships, the expression patterns, and the statistical characteristics of intronless genes. In addition, useful tools such as an advanced search and local BLAST are available through a user-friendly and intuitive web interface.

**Conclusion:**

In conclusion, the IGDD provides a comprehensive and up-to-date platform for researchers to assist the exploration of intronless genes in dicot plants.

## Background

Genes that are not interrupted by introns are called intronless genes or single-exon genes. Depending on their structure, genes in eukaryotes fall into two categories: intronless genes and intron-containing genes. Intronless genes can serve as beacons in analyses of gene function and evolution. For example, intronless genes as a model, compared with intron-containing homologs, can enable an inverse approach to studying the considerable roles of introns, which are only found in eukaryotes [1]. Furthermore, studies on intronless genes should help to unravel some evolutionary issues including: (i) the major factors that have contributed to the emergence of intronless genes (gene duplications, inheritance from ancient prokaryotes, retroposition or other mechanisms); (ii) the evolutionary significance of retroposition (retrogenes are considered to be intronless); and (iii) the biological origins of introns (i.e., which hypothesis is more correct: the introns-early hypothesis or introns-late hypothesis?) [2]. Therefore, related studies and the construction of databases for intronless genes have received great attention in recent years.

IGD [3] and PIGD [4] are two existing intronless gene databases that contain information about intronless genes in human and Poaceae, respectively. The identification methods and knowledge of intronless genes in these two databases are worthy of reference. However, limitation still persists as they still are not the centralized platforms for intronless genes. Furthermore, IGD is derived from GenBank, which does not contain all the sequences available from genome projects; thus, it does not provide representative intronless gene sets. Compared with IGD, PIGD has an improved methodology, but contains less evolutionary information in its gene annotations.

To build a centralized platform, we developed IGDD—a comprehensive and integrated database for intronless genes that covers five dicots, including *Arabidopsis thaliana* (Arabidopsis), *Carica papaya* (papaya), *Populus trichocarpa* (poplar), *Salix suchowensis* (willow) and *Vitis vinifera* (grape). These plants were chosen as representatives not only because of their burgeoning sets of genome sequencing, but also because of their obvious patterns of genome duplication [5]. Specifically, the *Arabidopsis thaliana* genome has undergone two whole genome duplication events (α and β) within the crucifer lineage and one more ancient genome triplication event (γ) shared with most dicots [6], *Carica papaya* and *Vitis vinifera* have each experienced only the γ triplication and no subsequent polyploidies; whereas *Populus trichocarpa* and *Salix suchowensis* have undergone a salicoid-specific duplication (ρ) besides the γ triplication [7]. The large number of paralogous genes present in these species offers a unique opportunity to help us elucidate the evolutionary consequences of intronless genes.

IGDD not only contains comprehensive knowledge and links to authoritative external datasets, but also includes the important features within and among species. In addition to basic information on intronless genes, the annotations in IGDD include predicted protein domains, KEGG pathways, GO items, functional descriptions and subcellular localizations. Moreover, IGDD also lists paralogs, orthologs, syntenic blocks, and expression patterns across different developmental stages and tissues. By integrating massive functional and evolutionary information and developing valuable tools, we aim to provide a useful resource and versatile platform that will benefit the related research community.

## Construction and content

### Data source

The genome sequences of Arabidopsis, papaya, poplar, and grape were acquired from the publicly available database Phytozome [8–11]. In our database, the latest versions of data in use are TAIR10 for *Arabidopsis*, ASGPBv0.4 for papaya, v3.0 for poplar, and Genoscope.12X for grape separately. The genome of *Salix suchowensis* is a most updated in-house database developed by our research group [7].

### Identification of intronless genes

Intronless genes were identified by Perl scripts. First, the scripts extracted the information for mRNAs that were not interrupted by “introns” from a GFF file, and genes containing only one continuous exon were considered putative intronless genes. Subsequently, the longest transcript was retained if two or more transcripts represented a gene that met the criteria in the first step. Considering their biological functions, only proteins encoded by intronless genes with lengths ≥ 30aa were selected.

### Annotation of intronless genes

To provide informative clues for further functional analysis, we systematically annotated each intronless gene. Initially, we obtained the standard gene information from Phytozome [12], including PFAM [13], GO annotation [14] and PANTHER data [15]. Functional description was then added using the InterproScan program (Version 5.44.0) [16]. Pathway annotations from the KEGG database [17] were also included in IGDD. In addition, we added subcellular localization prediction information from WoLF PSORT [18].

### Putative homolog annotation

To predict paralogs of the intronless genes, the method described by Blanc and Wolfe was used [19]. We performed all-against-all nucleotide sequence similarity searches among the transcribed sequences in each genome using the BLASTN software [20]. Sequences with strict cutoffs of alignment length ≥ 300 bp and identity ≥ 40 % were defined as paralogs. We also downloaded the paralogs of intronless genes annotated in Ensembl using Biomart [21]. The results obtained from both methods were included for accurate prediction of paralogs. Orthologs of intronless genes across the five dicots were identified by OrthoMCL with the default parameters [22].

### Syntenic analysis

To detect intronless genes that had arisen from whole-genome duplication (WGD), syntenic blocks within and between species were identified using BLAST, OrthoMCL and the MCscanX software [20, 22, 23]. We then retrieved the syntenic blocks containing intronless genes. Finally, to show an overview of all blocks, the intra-genome and inter-genome syntenic relationships of the intronless genes were visualized with Circos [24]. The nonsynonymous (Ka) and synonymous (Ks) substitution rates of gene pairs in each block were determined by KaKs_Calculator 2.0 [25].

### Expression patterns of intronless genes

An expression-based resource can provide an important bridge between genotype and phenotype through transcript profiles; therefore, we integrated a wide variety of expression data to explore the spatial and temporal expression patterns of intronless genes during development. Comprehensive microarray data for *A. thaliana* was acquired from TAIR across 63 tissues [26], for *P.trichocarpa* from NCBI-GEO (GSE13990) covering nine tissues [27], and for *V.vinifera* from NCBI-GEO (GSE36128), which included 54 samples representing green and woody tissues [28]. Expression data for *S. suchowensis* was obtained using RNA-Seq from five tissues [7]. The expression data of the intronless genes was imported into R and Bioconductor, and then the pheatmap package was used to make heatmaps.

## Utility and discussion

**Fig. 1:**
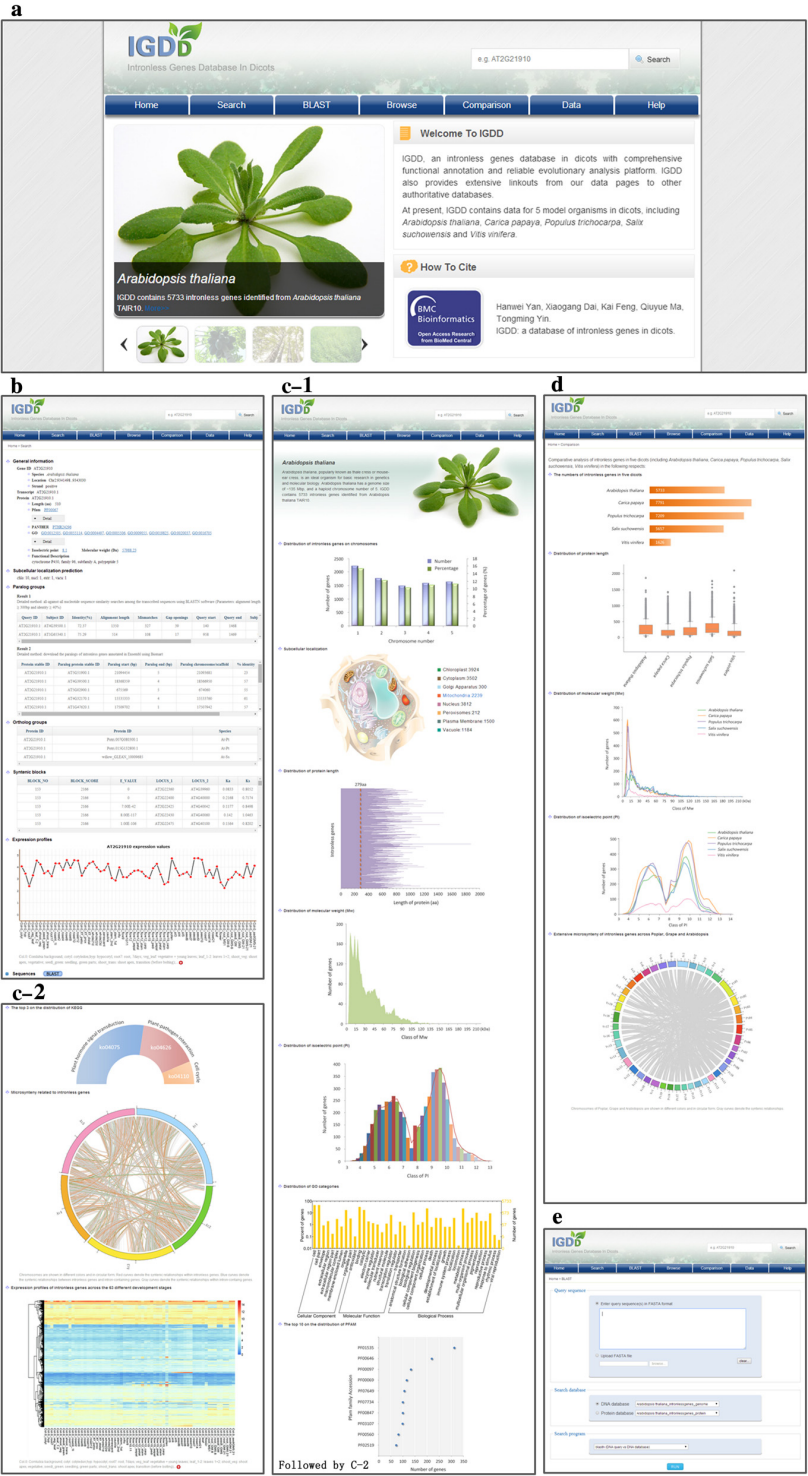
An overview of the IGDD website. **a** The home page. **b** Comprehensive individual gene information (e.g., AT2G21910). **c** The browse page. **d** The comparison page. **e** The BLAST page

IGDD collects sequence information from multiple resources to enrich the data for intronless genes. At present, IGDD contains 28,016 intronless genes from five dicots (Table 1). In IGDD, different categories of datasets are classified and compared within and among species with user-friendly graphics. Additionally, analytical tools such as BLAST are embedded in IGDD to help predict the putative orthologous groups of the intronless genes. Thus, IGDD is a solid web platform for searching, browsing, visualizing and downloading intronless genes (Fig. 1a). Below, we discuss the four main functional units in IGDD: (1) the comprehensive individual gene information, (2) the integration of data sources, (3) the BLAST sequence search engine, and (4) the interactive platform.

**Table 1:** List of five dicots currently served by IGDD

Species	Release version	Identified Intronless Genes	Percentage of Intronless Genes
*Arabidopsis thaliana*	TAIR 10	5733	20.9 %
*Carica papaya*	ASGPB V0.4	7791	28.1 %
*Populus trichocarpa*	V3.0	7209	17.4 %
*Salix suchowensis*	V 1.0	5657	21.3 %
*Vitis vinifera*	Genoscope.12X	1626	6.2 %

1. Comprehensive individual gene information
  IGDD provides detailed annotations for every putative intronless gene (Fig. 1b). Users can access the webpage in multiple ways, such as by clicking “Browse” on the main navigation bar or importing a gene name into the “Search” section directly. The annotation information for individual genes includes: (i) basic information, (ii) protein sequence features, (iii) homolog groups including paralogs and orthologs, (iv) syntenic blocks, and (v) expression profiles. In detail, basic information consists of the gene identifier, location, strand and annotations for the corresponding coding protein (such as length, isoelectric point (PI), and molecular weight (Mw)). The protein sequence features display protein identities (IDs) for PFAM, GO, PANTHER and KEGG, and functional descriptions. These IDs are hyperlinked to external databases to access more details. Additionally, subcellular localization prediction is shown on the page because it gives an important clue to the protein’s role(s). Gene duplication followed by rapid sequence divergence between paralogous pairs is considered to be the major mechanism for the emergence of new genes [29]. To determine the contribution of gene duplication to the amplification of intronless genes, we provide information on paralogous pairs among intronless genes, and between intronless genes and intron-containing genes. To explore cross-species evolutionary study, ortholog groups between intronless genes were identified. Among the syntenic blocks shown on the webpage, the corresponding intronless gene is highlighted in blue font to facilitate searching. The Ka and Ks values of paralogous pairs are also provided to determine their evolutionary distances. Knowing the expression patterns of intronless genes in different developmental stages and tissues is essential to illustrate whether they have corresponding biological functions. In IGDD, we extracted microarray and RNA-seq data to assess the temporal and spatial expression of intronless genes. When an intronless gene is found to be expressed, the expression data is visualized by a line diagram that allows users to view the expression changes across different tissues and developmental stages.
2. Integration of data sources
  Based on the collection of available data as aforementioned, individual genes were further analyzed and compared within and among species. We dedicated two main functions of the interface to this: the “Browse” and “Comparison” sections. Specifically, the comparative data for intronless genes within species was analyzed from a variety of perspectives, and visualized on the “Browse” webpage (Fig. 1c). From the interface, basic information on the classification under different attributes is displayed, such as the distribution of intronless genes on chromosomes, subcellular localization, distribution of protein length, PI and Mw. In particular, users can browse and download detailed data by clicking the corresponding section of the figure to view the differences. Apart from basic information, we also collected functional annotations through GO associations, PFAM domain information, and KEGG data. The “Browse” page shows the proportion of genes and gene product attributes associated with cellular components, biological processes, molecular functions, gene families, and pathways. To study genome organization and evolution, colinearity information within species can be applied to analyze segmental and WGD events. The Circos software [24] was employed to enable browsing of the syntenic relationships between intronless genes and other genes using different colored curves. In addition, to better understand whether intronless genes are associated with plant phenotypes, the gene expression in different tissues is clearly visible from the heat map. The heat map provides not only an overview of the global gene expression trends, but also conclusive evidence that these intronless genes are truly transcribed to mRNA.
  The “Comparison” section compares gene characteristics across species and provides detailed information by clicking on the image (Fig. 1d). We found that proportions of intronless genes against the total number of genes differed significantly across the five investigated species, with 20.9, 28.1, 17.4, 21.3, and 6.2 % in Arabidopsis, papaya, poplar, willow, and grape, respectively (Chi-square test, *P* < 0.05). The average protein sizes were 279, 182, 229, 333, and 178aa in Arabidopsis, papaya, poplar, willow, and grape, respectively. In pairwise comparisons, the differences in the average protein sizes were remarkable (μ-test; *P* < 0.05) except between papaya and grape. Conversely, we noticed that intronless genes had similar characteristics across the five dicots. For the protein features, the peak Mw value ranged from 8 to 13 kDa, and the PI distribution showed two main peaks at 6 and 9.
3. The BLAST sequence search engine
  The BLAST (Basic Local Alignment Search Tool) interface provides a flexible way to search for homologous genes of every intronless gene stored in IGDD. Users can submit query sequence(s) and adjust the BLAST parameters including database and search program. To make viewing the result page easier, we set the following two parameters: e-value cutoff of 1e-5 and retaining the top 100 hits based on e-values.
4. The interactive platform
  To expand the annotation scope of IGDD, reliable functional and evolutionary information was retrieved from the well-known databases PFAM, PANTHER, KEGG, GO and Expasy. Once plant genomics websites release the latest versions of the four dicots genomes, the datasets in IGDD would be updated synchronously. Compared with comparative genomics websites, IGDD focuses on the relationship among intronless genes, which is helpful to explore functional and evolutionary features on intronless genes across dicots. All of the information in IGDD is available for researchers at an FTP (File Transfer Protocol) site. In addition, IGDD encourages users to submit fully annotated intronless genes from other dicots to the database. When the quality of the submitted information meets our requirements, the IGDD curator will import the data into the database. Overall, the interactive platforms, including links to external databases, and download and submission components, make IGDD a comprehensive and systematic platform for the research community.

## Conclusions

Our main goal with IGDD is to construct a comprehensive platform for the exploration of intronless genes in dicots. IGDD integrates various types of data and links with multiple external databases to provide rich annotation information, which can be browsed and retrieved through user-friendly web interfaces. Biological tools such as BLAST and a comparison platform are also provided to facilitate investigations into the functional and evolutionary consequences of intronless genes. In future, the authors will regularly review authoritative databases for new dicot genomes, and expand the content of IGDD. Moreover, we intend to add new functions and integrate multiple data sources to enhance the IGDD database. For example, expression profiles will focus not only on different tissues and developmental stages, but also experimental treatments, such as biotic and abiotic stress. Our ultimate goal is to construct a co-expression network that provides informative clues to the regulation of intronless gene expression. In conclusion, we hope that IGDD will serve as a useful resource and fundamental platform for studying intronless genes, especially their occurrence and evolution.

## Availability and requirements

Database: IGDD

Database homepage: http://bio.njfu.edu.cn/igdd

Operating system(s): Unix

Programming language: C+, JavaScript, Perl

Other requirements: MySQL, Apache, PHP

These data are freely available without restrictions for use by academics. Inquiries concerning the database may be directed to IGDD@163.com.

## Abbreviations

IGDD, intronless genes database in dicots

WGD, whole-genome duplication

PI, isoelectric point

Mw, molecular weight

BLAST, basic local alignment search tool

FTP, file transfer protocol
